# VS-5584 mediates potent anti-myeloma activity via the upregulation of a class II tumor suppressor gene, RARRES3 and the activation of Bim

**DOI:** 10.18632/oncotarget.21988

**Published:** 2017-10-20

**Authors:** Nurulhuda Mustafa, Jeannie Xue Ting Lee, Huey Fang Adina Nee, Chonglei Bi, Tae-Hoon Chung, Stefan Hart, Wee Joo Chng

**Affiliations:** ^1^ Yong Loo Lin School of Medicine, National University of Singapore, Singapore; ^2^ Cancer Science Institute of Singapore, National University of Singapore, Singapore; ^3^ Department of Haematology-Oncology, National University Cancer Institute of Singapore, National University Health System, Singapore; ^4^ S*BIO Pte Ltd., Singapore

**Keywords:** myeloma, dual PI3K/mTOR inhibitor, RARRES3, panobinostat, therapeutics

## Abstract

The PI3K/mTOR/AKT pathway is an integral regulator of survival and drug resistance in multiple myeloma (MM). VS-5584 was synthesized with dual-specific and equipotent activity against mTORC1/2 and all four Class I PI3K isoforms so as to durably inhibit this pathway. We show that VS-5584 is highly efficacious against MM cell lines even in the presence of IL-6 and IGF-1 and that this growth inhibition is partially dependent on Bim. Importantly, VS-5584 triggers apoptosis in patient cells with a favorable therapeutic index. Gene expression profiling revealed a VS-5584-induced upregulation of RARRES3, a class II tumor suppressor gene. MM patient databases, UAMS and APEX, show that RARRES3 is under-expressed in 11q13 subsets which correlates with the reduced effectiveness of VS-5584 in 11q13 cell lines. Silencing RARRES3 expression significantly rescues VS-5584-induced cell death and increases cyclin D2 expression but not cyclin D1 or other cyclins implying a role for RARRES3 in cell cycle arrest. In vivo, VS-5584 significantly reduces the tumor burden of MM mouse xenografts. We further identified that VS-5584 synergised with Dexamethasone, Velcade, and exceptionally so with HDAC inhibitor, Panobinostat. Interestingly, this was consistently observed in several patient samples, proposing a promising novel clinical strategy for combination treatment especially in relapsed/refractory patients.

## INTRODUCTION

The malignant proliferation and pathophysiology of multiple myeloma (MM) cells is driven by genetic aberrations in the cell and sustained by the rich signaling input derived from the interaction between the myeloma cells and the bone marrow microenvironment (BMM). The PI3K/Akt/mTOR pathway is central to this complex signaling network and can be found constitutively active in MM cell lines as well as bone marrow biopsies of MM patients [1]. It is the major pathway stimulated by bone marrow growth factor cytokines IL-6 and IGF-1 and has been shown to play a key role in mediating MM proliferation, survival and cell death [1, 2]. PTEN negatively disrupts this signaling pathway by dephosphorylating PIP3 to PIP2. When PTEN null or PTEN overexpressing myeloma cells were injected into SCID mice, all PTEN null mice developed tumors, while only 50% of those injected with PTEN overexpressing MM cells developed tumors, which were additionally both smaller and required longer than 5 times the latency period of controls for formation [3]. Interestingly, further evidence for the importance of this pathway in MM has also been shown by Du et al. where the disruption of PI3K/mTOR/Akt pathway negatively modulates the survival of the myeloma cancer stem cell fraction [4]. Cancer stem cells (CSC) which persist in bone marrow niches have been attributed to relapsed or refractory myeloma disease. In this study, the authors reported that by targeting the mir-451-mediated activation of the PI3K/mTOR/Akt pathway with inhibitors Rapamycin and S14161, the myeloma CSC population was directly reduced via apoptosis [4]. Taken together, these evidence demonstrate the potential for inhibiting the PI3K/mTOR/Akt pathway for the effective and persistent retardation and prevention of *in vivo* growth of MM.

So far, single-target inhibitors of the PI3K/mTOR pathway such as Rapamycin and Perifosine have not shown promising clinical response rates [5]. This is in part due to the reactivation of Akt through inhibition of the negative feedback pathway exerted by TORC1 as well as the due to the heterogeneous nature of MM [6]. In addition, some rapalogs are unable to inhibit both TORC1 and TORC2 complexes resulting only in partial inhibition of the pathway. While there are indeed mTOR selective inhibitors that can target both TORC1 and 2, resistance continues to develop as a product of the PI3K feedback mechanism mediated by an upregulation of Akt phosphorylation or via Akt-independent downstream PI3K effector targets [7].

VS-5584 is a highly selective purine analog, equally potent against PI3K (all 4 isoforms of catalytic subunit p110 (α, β, γ, δ)) and both mTORC1 and 2 resulting in the robust inhibition of the phosphorylation of the downstream substrates of these targets. This enables it to overcome the effects of PI3K-feedback signaling mechanisms as well as mitigates the upregulation of the Ras-MAPK pathway that is observed after mTORC1 only inhibition [8]. To date, there have been several pre-clinical reports of dual PI3K/mTOR inhibitors including PI-103, XL765, and NVP-BEZ235 [1, 5, 9–12] [13, 14]. PI-103 did not enter clinical trials due to issues of rapid metabolism *in vivo* and NVP-BEZ235 is currently in several phase I/II trials for renal and prostate cancer [15]. VS-5584 is differentiated from similar clinical staged compounds by targeting mTOR and class I PI3K in the same low IC_50_ range while evincing no effects on more than 400 other lipid and protein kinases tested [8]. It exhibits favorable pharmacokinetic properties after once daily oral dosing in mice where mTORC1 and 2 and PI3K signaling were effectively abrogated in tumor tissues of prostate cancer and AML xenograft models. VS-5584 shows inhibitory activity across a broad range of tumor types. Strikingly, multiple myeloma cell lines showed highest sensitivity to the drug in comparison to bladder, breast colorectal, prostate cancer and leukemia cell lines [8]. In this study, we evaluated the efficacy of VS-5584 in MM and investigated the underlying mechanism mediating its anti-myeloma effects.

## RESULTS

### VS-5584 inhibits PI3K/mTOR/Akt pathway signaling in MM

**Figure 1:**
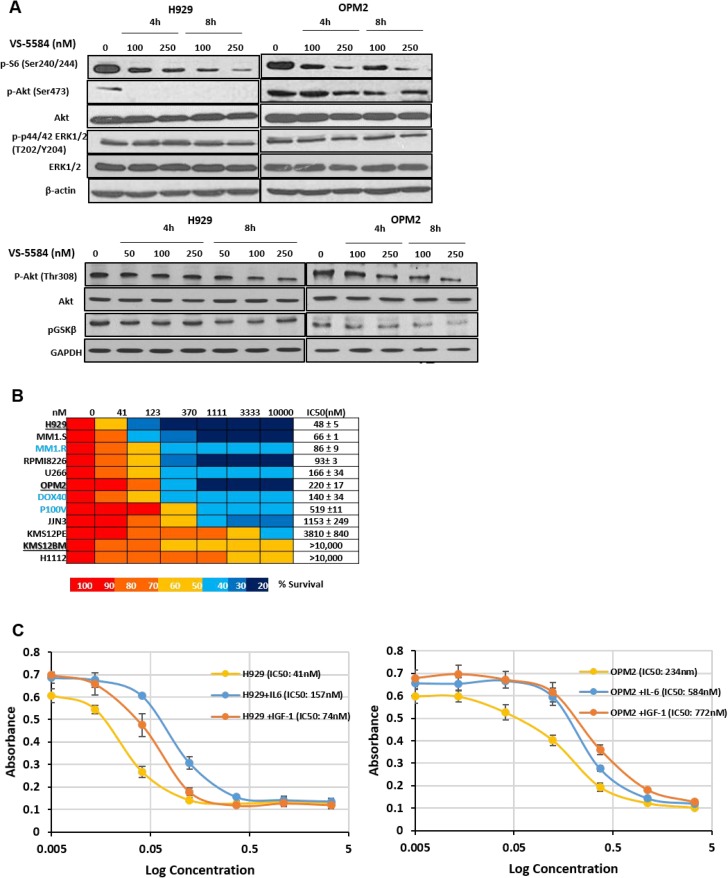

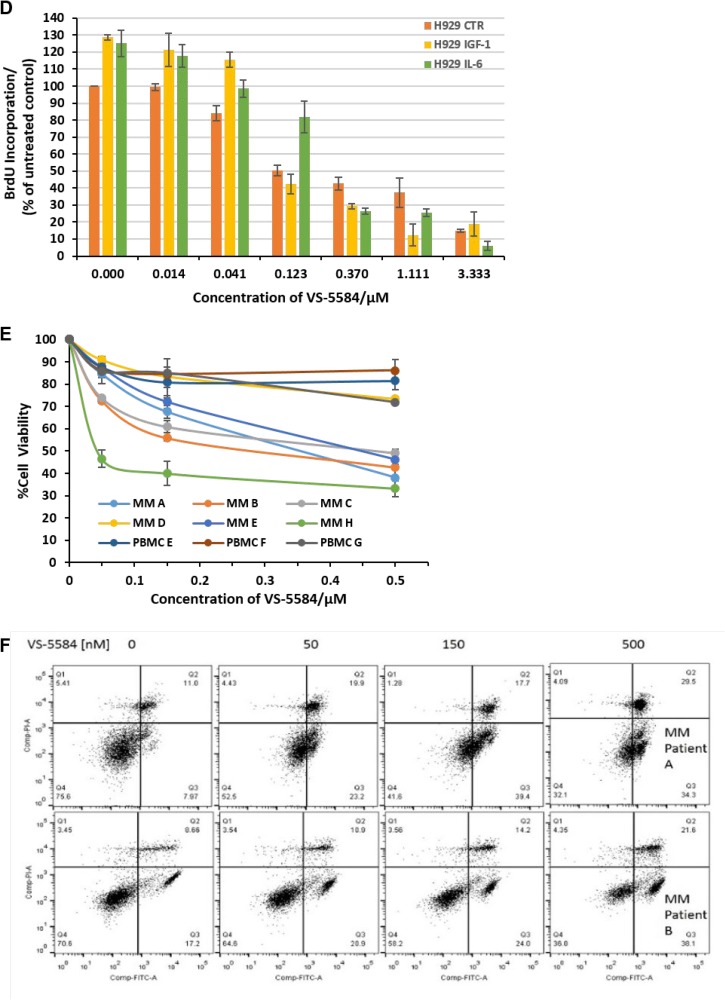
VS-5584 effectively suppresses PI3K and mTOR signaling pathways and significantly inhibits viability of MM cell lines as well as patient samples at low concentrations while overcoming protective effects of growth cytokines (**A**) H929 and OPM2 cell lines were treated with VS-5584 for 4 h and 8 h at concentrations as indicated. After cell lysis the phosphorylation status of pS6, pAkt, pGSKβ and p44/42 MAPK (ERK1/2) were detected by immunoblotting. (**B**) MM cell lines were treated with VS-5584 (0–10 μM) and assayed by MTS at 48h. Most of the cell lines are sensitive with an IC50 < 500 nM. KMS12PE, KMS12BM and H1112 were least sensitive with an IC50 of > 4μM. (**C**-**D**) MM cells are supplemented with IL-6 (10 ng/mL) or IGF-1 (50 ng/mL), treated with VS-5584 for 48h and then viability was assessed with (C) MTS (Promega) or (D) BrdU Proliferation Assay. Graphs are representative of 3 different experiments performed in triplicate. (**E**) CD138 + cells isolated from bone marrow aspirates of MM patients or PBMCs stimulated with 5ng/mL of PHA were treated with VS-5584 for 48h. Cell viability was measured with MTS (Promega). (**F**) CD138 + MM patient isolates were exposed to 50 nM, 150 nM and 500 nM of VS-5584 for 48h. Subsequently cells were stained with Annexin V FITC and PI and analysed using flow cytometry. At least 10,000 events were analysed where *n* = 3.

Western blot analysis confirms the dual inhibitory activity of VS-5584. It showed that the protein levels of the substrates of (1) the PI3K pathway- phospho-Akt (Thr308) and phospho-GSKβ as well as substrate of (2) the mTORC2 pathway phospho-Akt (Ser473) and (3) mTOR/AKT substrate, phospho-S6 have been attenuated by VS-5584 treatment. Expression levels of phosphorylated Akt (Ser473) were completely abolished in H929 (hypersensitive) and reduced in OPM2 (less sensitive) (Figure 1A). Phospho-Akt(Thr308), phospho-GSKβ and Phospho-S6 ribosomal protein expression levels were similarly downregulated, albeit requiring a higher concentration of VS-5584. Additionally we observe no significant change in the levels of phospho-p44/42-MAPK, thus verifying the specific targeting of VS-5584 on the PI3K/mTOR/Akt signaling pathway.

### Low concentrations of VS-5584 is cytotoxic in MM cell lines and is able to overcome protective effects of MM growth cytokines

To investigate the effect of VS-5584 on growth and survival, a wide panel of representative MM cell lines were treated with VS-5584 in the range of 0–10μM for up to 48h and cell viability analyzed with the MTS assay. Overall, VS-5584 strongly inhibited cell viability in all MM cell lines (Figure 1B) including cell lines which are resistant to the drugs Doxorubicin, Dexamethasone and Velcade. It was most efficacious in H929 (IC_50_ = 48nmol/L) with IC50 still remaining in nanomolar range in the Velcade-resistant cell line. In our panel we identified 2 cell lines KMS12BM and H1112 which seemed rather refractory to drug with about 50% cell death upon treatment with the highest concentration tested at 10μM.

We further studied the effect of VS-5584 treatment on the substrates of PI3K/AKT/mTOR pathway in the refractory cell line KMS12BM and discovered that VS-5584 was able to significantly inhibit phospho-Akt (Thr308), phospho-GSKβ, phospho-Akt (Ser473) and phospho-S6 at the relatively low concentrations of 250 and 500nM (Supplementary Figure 1A). However at these concentrations, KMS12BM was still 60–70% viable suggesting therefore that a downstream component regulating cell survival or cell death is most likely a factor contributing to the refractory response.

In multiple myeloma, growth factor cytokines such as IGF-1 and IL-6 mediate a complex signaling network which stimulates the proliferation of MM cells. H929 and OPM2 were co-cultured with exogenously provided IL-6 (10 ng/mL) or IGF-1 (100 ng/mL) and then treated with VS-5584. However, the supplemented cytokines did not provide significant survival benefit to the cell lines (Figure 1C) and the IC50 of the cells remained in the nanomolar range. Figure 1D confirms that VS-5584 reduces BrdU incorporation and thus inhibits cytokine-mediated cell proliferation.

### VS-5584 treatment targets primary patient cells to induce apoptosis and displays a favorable therapeutic index

Purified CD138^+^ MM cells were harvested from the bone marrow aspirates of MM patients and treated with VS-5584 up to a concentration of 500nM for 48h. In a cell viability assay, 5 out of 6 patient samples showed sensitivity to VS-5584 with IC_50_ values in the range of 50nM-500nM (Figure 1E). Importantly, VS-5584 showed negligible toxicity at the same concentrations in PBMCs thus proposing a favorable therapeutic index. We subsequently investigated if the inhibition in cell viability was a function of apoptosis. We indeed observed a dose-dependent increase in the Annexin V+PI- staining (Figure 1F) in patient cells.

### VS-5584 stimulates G1 arrest followed by the induction of apoptosis which is dependent on pro-apoptotic Bcl-2 family protein Bim

**Figure 2:**
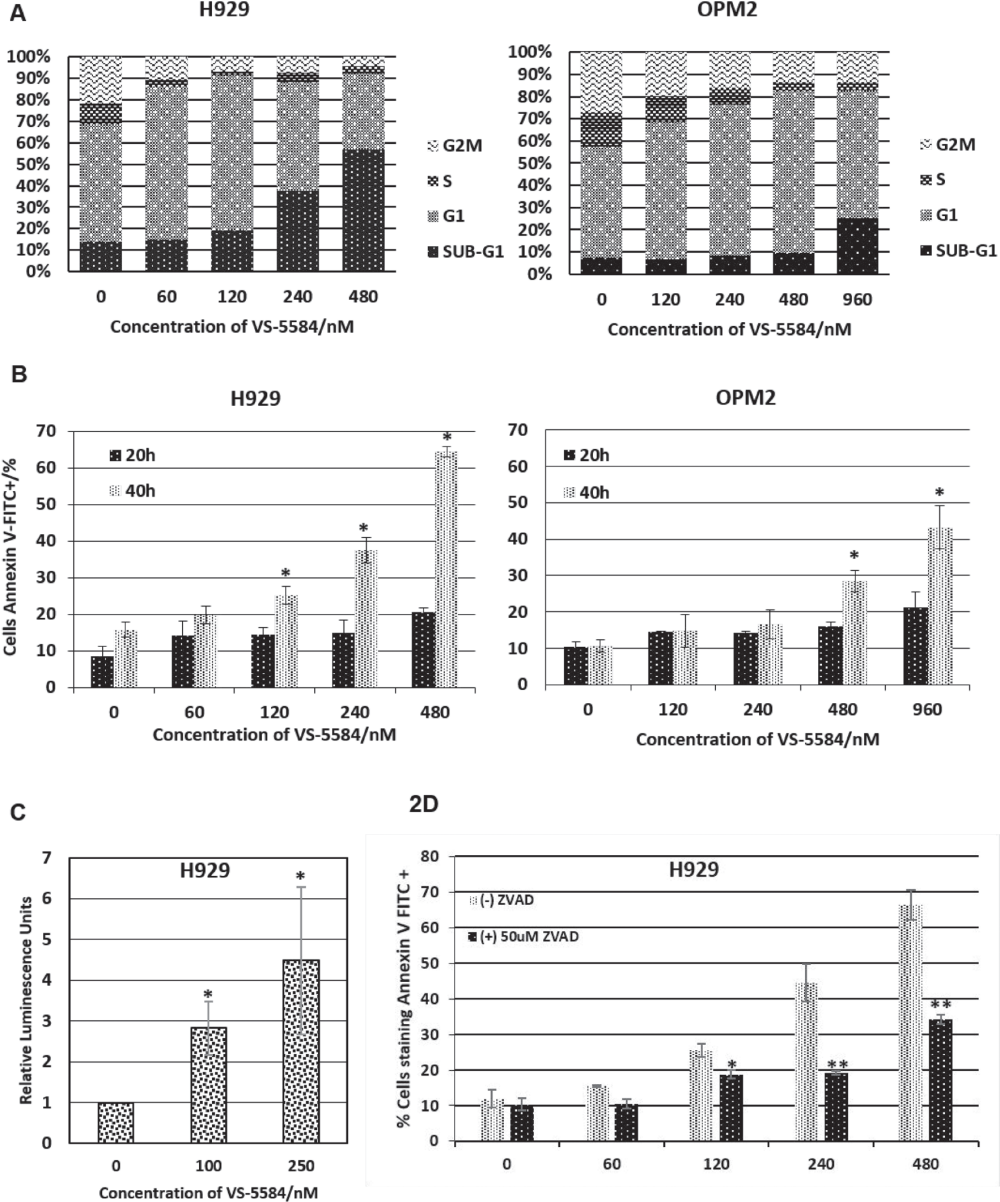
VS-5584 targets MM cells to apoptosis (**A**) H929 and OPM2 were treated with VS-5584 at the respective concentrations and harvested at 48 hours. They were subsequently fixed and permeabilised with ethanol and then stained with PI. At least 10,000 events were analysed (**B**) H929 and OPM2 cells were treated with the stated concentrations of VS-5584. At 20 and 40h the cells were subsequently harvested and stained with Annexin V FITC and PI and analyzed by flow cytometry. The graph is a representation of Annexin V positive cells,^*^p < 0.05. (**C**) Caspase 3 is activated. Cells were treated with VS-5584 for 48 hours and then lysed and incubated with a substrate of caspase 3 which luminesces upon cleavage by caspase 3. This graph shows the fold increase in luminescence relative to untreated control, ^*^p < 0.05. (**D**) H929 cells were pre-incubated with 50 μM of zVAD-fmk(Santa Cruz) for 1 h before being treated with VS-5584 for 48h. The cells were subsequently harvested, stained with Annexin V FITC and analysed by flow cytometry where at least 10,000 events were analysed, ^*^p < 0.05, ^**^p < 0.01.

To further understand the mechanism for the induction of apoptosis triggered by VS-5584 we studied the cell cycle profile of H929 (hypersensitive) and OPM2 (less sensitive) cells during drug treatment. Interestingly, both cell lines showed identical trends of the induction of G1 arrest followed by an increase in sub-G1 population which is an indication of fragmented DNA formed during apoptosis (Figure 2A). We confirmed the induction of apoptosis by assessing for increased phosphatidylserine externalization. Our studies show that there was indeed a dose and time dependent increase in Annexin V^+^ staining induced by the treatment of VS-5584 in both H929 and OPM2 (Figure 2B).

Caspase 3 is the classical mediator of a caspase-dependent apoptotic cell death. A luminescence-based caspase 3 activity assay showed a dose-dependent increase in caspase 3 activity induced by VS-5584 which further corroborates with the induction of apoptosis (Figure 2C). Additionally, the cells were pre-treated with a pan-caspase inhibitor zVAD-fmk to confirm the involvement of a caspase-dependent apoptotic cell death (Figure 2D). Pre-treatment with the pan-caspase inhibitor is able to significantly rescue VS-5584 induced apoptosis.

The initial cell viability data had identified a differential response of MM cell lines to VS-5584 treatment where H929 was most sensitive and KMS12BM and H1112 were most refractory to the drug. To uncover potential mechanistic factors that may contribute to this response, we analyzed expression levels of pro and anti-apoptotic proteins in VS-5584 treated H929 (hypersensitive), OPM2 (less sensitive) and KMS12BM cells (refractory).

**Figure 3:**
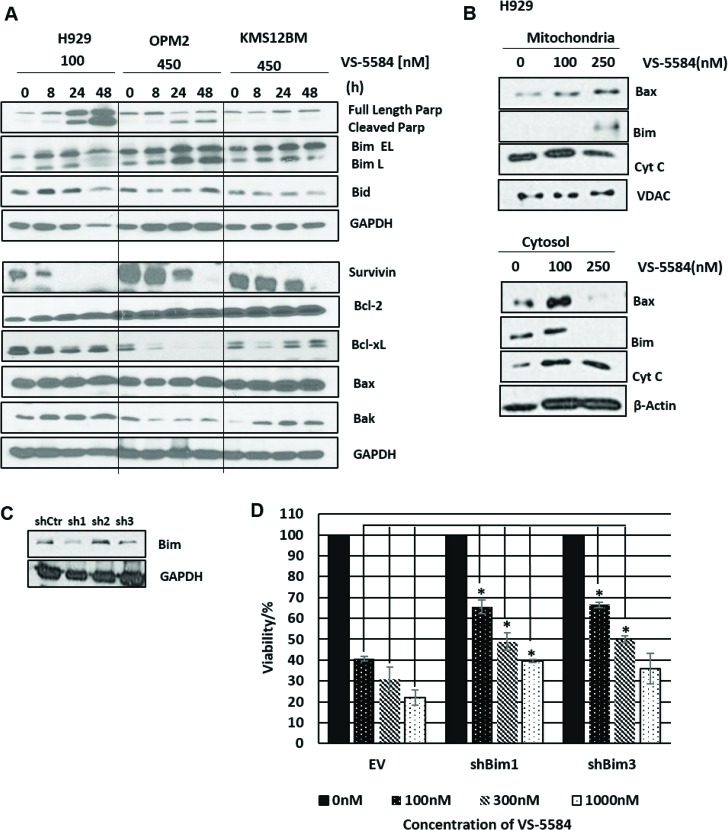
VS-5584 activates pro-apoptotic factors in the mitochondria (**A**) H929, OPM2 (sensitive) and KMS12BM (‘refractory’) were treated with VS-5584 at the following concentrations and harvested at 0,8,24 and 48 h. Subsequently cells were lysed and immunoblotted for apoptotic markers cleaved PARP as well as anti-apoptotic proteins (survivin, Bcl-2 and Bcl-xL) and pro apoptotic proteins (Bim, Bid, Bax and Bak) and a representative western blot was shown. (**B**) H929 was treated at the respective concentrations for 48 hours and subsequently lysed. We performed a sub cellular fractionation to separate the cytosolic and mitochondrial components of the cell and subsequently immunoblotted for Bax, Bim and Cytochrome C. (**C**) To investigate the role of Bim, MM1.S cells were infected with 3 different shRNAs for Bim. Cell lysates were subsequently immunoblotted for expression of Bim. (**D**) MM1.S cells were infected with shCtr, BimshRNA1 and BimshRNA3 and then subsequently treated with VS-5584 at the respective concentrations. At 48h cell viability was measured. This graph is representative of one experiment, ^*^p < 0.05 All experiments were performed where *n* = 3.

Firstly, poly (ADP-ribose) polymerase (PARP) cleavage was observed only in H929 and OPM2 but not KMS12BM which is consistent with earlier cell viability data (Figure 3A). There did not seem to be a consistent and significant effect on global expression levels of Bax, Bid, Bak and Bcl-2. There was however a significant increase in the expression of pro-apoptotic BH3 proteins Bim EL and Bim L as well as a significant downregulation of anti-apoptotic proteins survivin and Bcl-xL (Figure 3A). Bim was more significantly increased in H929 and OPM2 as compared to KMS12BM and Bcl-xL was only downregulated in H929 and OPM2 but not KMS12BM.

In addition we found evidence for the involvement of the intrinsic apoptotic pathway. Our investigation showed the translocation of Bax from the cytosol into the mitochondria (Figure 3B). This is congruent with the activation of the intrinsic apoptotic pathway which requires the homodimerization of Bax at the mitochondrial membrane to facilitate the release of pro-apoptotic factors from the intermembranal space into the cytosol. The western blot detected the release of Cytochrome C into the cytosol which is a pre-requisite for the cleavage of caspase 9. Interestingly we also observed the translocation of Bim from the cytosol into the mitochondria where Bim is known to bind to and antagonize anti-apoptotic members of the Bcl-2 family (Figure 3B).

As with H929 and OPM2, treatment with VS-5584 in MM1.S significantly suppressed the PI3K/mTOR/AKT pathway, accompanied with a concomitant upregulation in Bim and apoptosis (Supplementary Figure 1B). In order to clarify the importance of Bim in mediating VS-5584 induced cell death, MM1.S cells were infected with lentiviruses containing shRNA of Bim (Figure 3C). Our results show that when the expression of Bim is suppressed, VS-5584 is unable to efficiently inhibit cell viability in MM cells, implying a Bim-dependent induction of apoptosis (Figure 3D).

### RARRES3 (Retinoic acid receptor responder 3) contributes to the regulation of cyclin D2 suggesting a role in mediating VS-5584 induced G1 arrest

**Figure 4:**
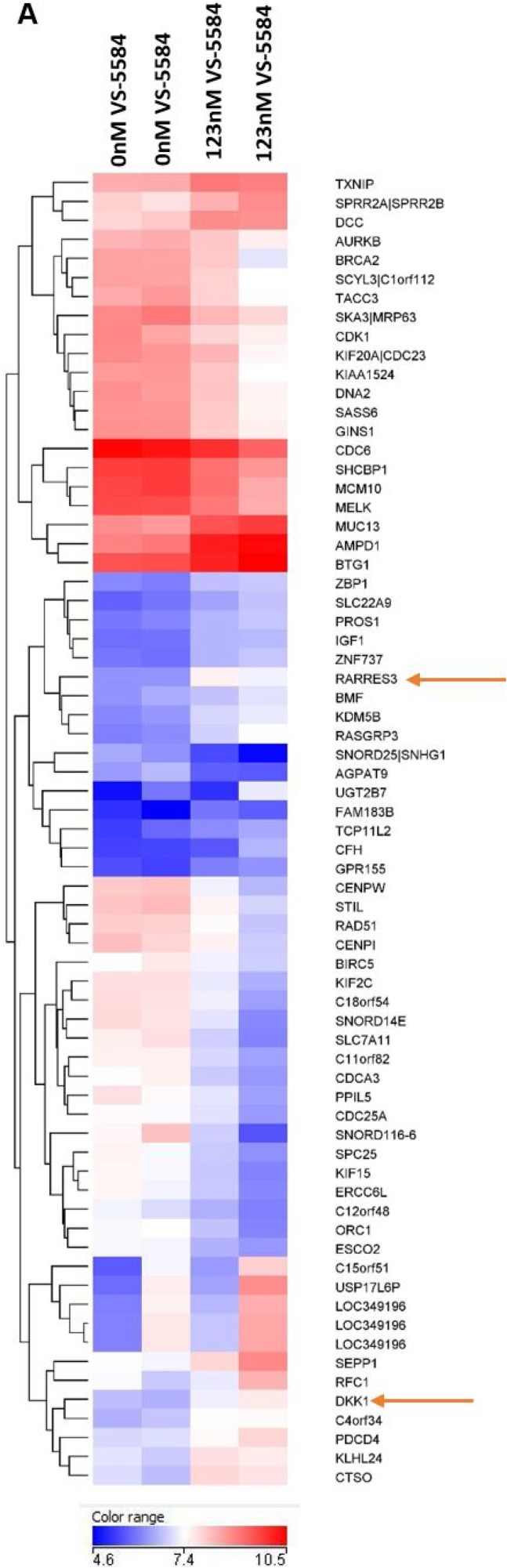

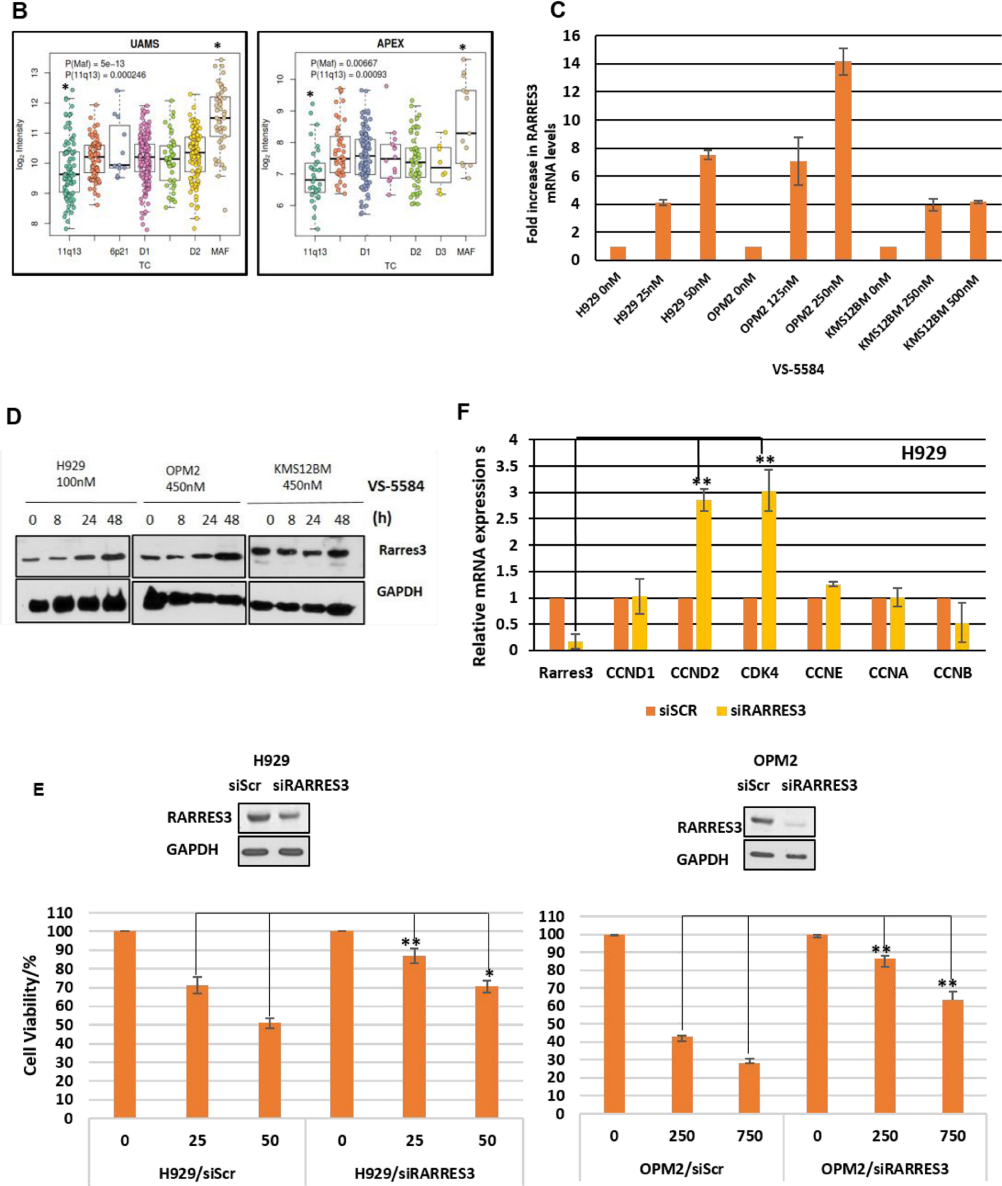
RARRES3 is an early response gene critical in mediating inhibitory effects of VS-5584 treatment (**A**) OPM2 cells were cultured in the presence or absence of 125nM of VS-5584 for 24 h which is half of the IC50 of OPM2 at 48 h. Total RNA was extracted and purified, cDNA synthesized and cRNA labelled prior to hybridization to the Affymetrix Human Gene 1.0 ST Array. The top 32 upregulated and top 38 downregulated genes are represented in the heat map according to the linear colour scale. (**B**) The MM patient GEP database UAMS and APEX were analyzed for any correlation between tumor suppressor gene RARRES3 and distinct clinical subsets of MM. (**C**) H929, OPM2 and KMS12BM cells were treated with indicated concentrations at 24 h and levels of RARRES3 mRNA measured via qPCR. (**D**) Cells were treated with indicated concentrations of VS-5584 over 0,8, 24 and 48 hours and RARRES3 protein expression levels monitored by immunoblotting. (**E**) RARRES3 siRNA or siScrambled were transfected into H929 and OPM2 cells via NEON electroporation for 24 h and the cells subsequently treated with VS-5584 at the indicated concentrations. Cell viability was measured after 48h ^*^p < 0.05, ^**^p < 0.01. These experiments are representative qPCR, western blot and cell viability analyses where *n* = 2 or 3 and each experiment is performed in triplicate. (**F**) RARRES3 was knocked down in H929 cells via NEON electroporation and mRNA was extracted. Levels of cell cycle proteins were measured with qPCR.

A finer understanding of the mechanism of action of a novel drug is invaluable in providing insights as to potential successful combinations or contraindications when the drug is applied in clinical trials. Thus, we performed a gene expression analysis on OPM2 treated with half IC50 concentration of the drug and harvested at 24h (before the 48h apoptotic induction) so as to identify early and sensitive genes that are critical in triggering VS-5584-induced cell death. The top 38 and 32 genes down and upregulated respectively by VS-5584 is shown in Figure 4A. Of particular interest was the RARRES3 gene which was the 2nd most upregulated gene in the list. RARRES3, is a class II tumor suppressor gene previously reported to be suppressed with disease progression in B cell lymphocytic leukemia due to its role in regulating cell cycle proliferation [16].

The role of RARRES3 is yet undescribed in the pathogenesis of MM. To give us an insight, we studied 2 different MM patient databases, the UAMS and APEX database for any correlations between the expression of RARRES3 with MM molecular subtypes. Interestingly, we found that the low expression of RARRES3 was significantly correlated with patients carrying the 11q13 MM subtype (Figure 4B). This seems to also correspond to the poorer efficacy of VS-5584 in 11q13 cell lines (KMS12BM and H1112).

Further investigation was warranted and first we validated the GEP analysis, by performing a qPCR to study the mRNA levels of RARRES3 present after treatment with VS-5584 (Figure 4C). There was a dose dependent increase in the expression of RARRES3 mRNA in H929 and OPM2 and a small increase in VS-5584-refractory KMS12BM. However, this increase in mRNA expression levels is translated to a corresponding increase in protein RARRES3 levels in VS-5584 sensitive cells, H929 and OPM2 but not in KMS12BM (Figure 4D).

To investigate the importance of the upregulation of RARRES3 by VS-5584, RARRES3 gene was silenced in H929 cells and the cells subsequently treated with VS-5584. Knocking down RARRES3 was able to significantly rescue the cell survival after drug treatment (Figure 4E). It was previously described that t(11;14) translocation subtypes contain cyclin D1 which is not responsive to dual PI3K/mTOR inhibition [17]. Additionally, RARRES3 has been reported to induce cell cycle arrest in some cancer cell lines [18]. We hypothesized that RARRES3 may play a role in regulating cyclins in MM. Thus after RARRES3 was knocked down, we also studied the mRNA expression levels of cyclins A, B, D1, D2 and E, as well as CDK4. We found that RARRES3 knock down led to the increase in expression of cyclin D2 but not cyclin D1 (Figure 4F). All the other cyclins also remained relatively unaffected. CDK4 which is the target of cyclin D, on the other hand showed a significant increase in expression. Thus, it seems VS-5584 treatment leads to the upregulation of the class II tumor suppressor RARRES3 which in turn downregulates Cyclin D2 and CDK4 thereby triggering cell cycle arrest. This suggests that the low expression of RARRES3 may contribute to the lack of efficacy of the drug in t(11;14) cells.

### MM cells are targeted to VS-5584 induced cell death partially via the inhibition of the β-catenin pathway

**Figure 5:**
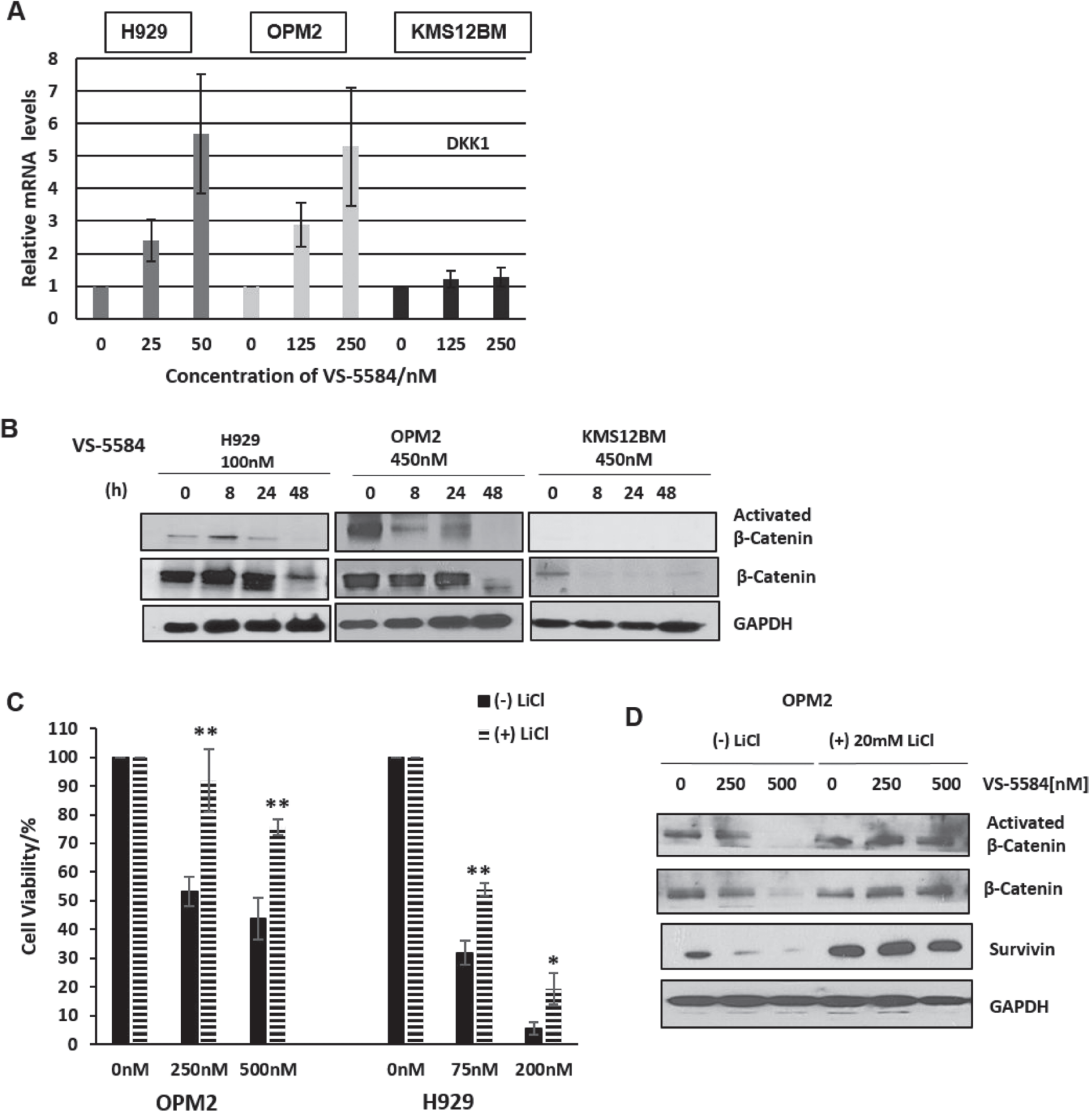
VS-5584 downregulates β-Catenin signaling (**A**) MM cells were treated at stated concentrations for 24h and harvested. Total RNA extracted and cDNA was synthesized and mRNA expression of DKK1 measured. (**B**) H929, OPM2 and KMS12BM were treated with 100nM and 450 nM of VS-5584 respectively and harvested at the indicated timepoints. Lysates were then immunoblotted for Activated β-catenin and β catenin. (**C**, **D**) H929 and OPM2 cells were pretreated with 10mM and 20 mM of LiCl respectively for 4 h and then treated with VS-5584 at the indicated concentrations. At 48 hours, cell viability was assessed with the (C) CTG assay and (D) levels of expression of β-Catenin activated β-Catenin and survivin were analyzed by immunoblotting. ^*^p < 0.05, ^**^p < 0.01.

Another interesting gene that was upregulated in the GEP analysis of drug-treated OPM2 cells was DKK1. DKK1 is a negative regulator of the Wnt/β-catenin pathway. In MM, the canonical Wnt/β-catenin signaling pathway is constitutively activated and is reportedly involved in cell cycle regulation, proliferation and invasion [19–21]. The upregulation of DKK1 suggests the potential involvement of the Wnt/β-catenin pathway in mediating the effects of VS-5584. Our qPCR validation study of the mRNA expression levels of H929, OPM2, KMS12BM cells treated with VS-5584 revealed an interesting and corroborative upregulation of DKK1 in H929 and OPM2 cells but not VS-5584 insensitive KMS12BM cells (Figure 5A). We further studied the levels of β-catenin in H929 and OPM2 and found that the active β-catenin was significantly downregulated by the treatment of VS-5584 (Figure 5B) but not in KMS12BM. Active β-Catenin was distinguished from total β-Catenin by specifically probing with an antibody that detects the protein only when it is not phosphorylated at Ser33/37 or Thr41. Lithium Chloride (LiCl), a β-catenin stabilizing compound, mimics Wnt signaling by inhibiting GSK-3β which prevents the degradation of β-catenin and thereby stabilizes its expression. Pre-treatment of the cells with LiCl before adding VS-5584 showed a drastic rescue in the cell death phenotype in both OPM2 and H929 cells (Figure 5C). Western blot analysis shows that pre-treatment with LiCl completely restored the expression levels of β-catenin as well as a downstream β-catenin target survivin (Figure 5D). This provides evidence that VS-5584-induced cell death in MM cells in part mediated via the inhibition of the β-catenin pathway.

### VS-5584 can inhibit tumor growth in a MM xenograft mouse model

**Figure 6:**
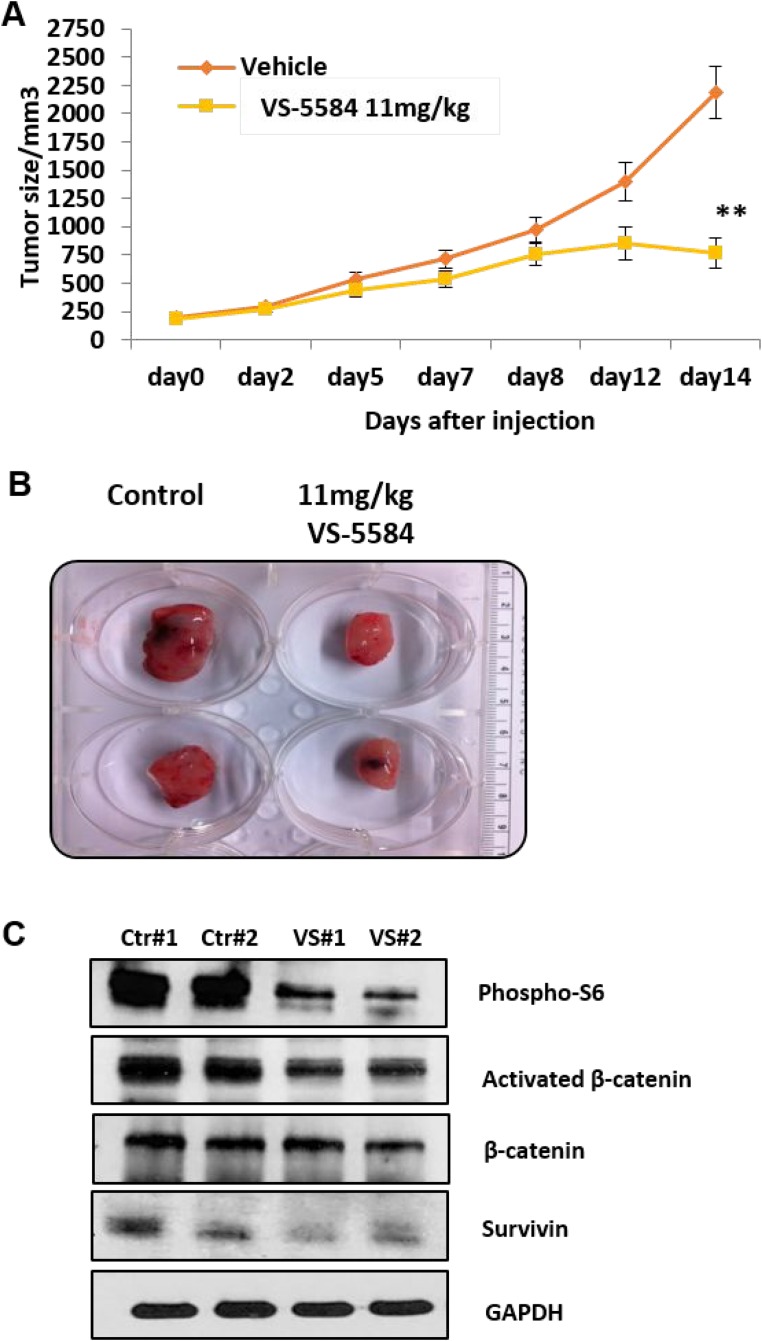
Oral administration of VS-5584 in a MM xenograft mouse is able to significantly inhibit tumor cell growth (**A**) NOD/SCID mice were implanted intra-dermally in the right flank with OPM2 cells. Dosing started after the tumor was palpable and the mice were administered an oral dose daily with VS-5584 or Vehicle for 5 days followed by 2 days rest (*n* = 6 in each group). The graph shows the analysis of the size of the tumor over time where ^**^p < 0.01 and (**B**) the image shows tumor harvested from mice treated with control or 11mg/kg of VS-5584. (**C**) Tumors were harvested after treatment and immunoblotting performed to analyze levels of phospho-S6, Activated β-catenin, β-catenin and survivin.

It is important to confirm the anti-tumor efficacy of a drug in an *in vivo* model in order to progress towards clinical evaluation. VS-5584 significantly reduced tumor burden as compared to controls (Figure 6A, 6B). No significant changes in weight or other signs of potential toxicity were observed. Immunoblotting analysis performed on tumors harvested from the mice showed that *in vivo*, VS-5584 was able to inhibit the downstream targets of PI3K/mTOR pathway phosphorylated-S6 as well as key mediators of cell inhibition active β-catenin and survivin (Figure 6C).

### VS-5584 synergizes with several MM clinical therapeutics

**Figure 7:**
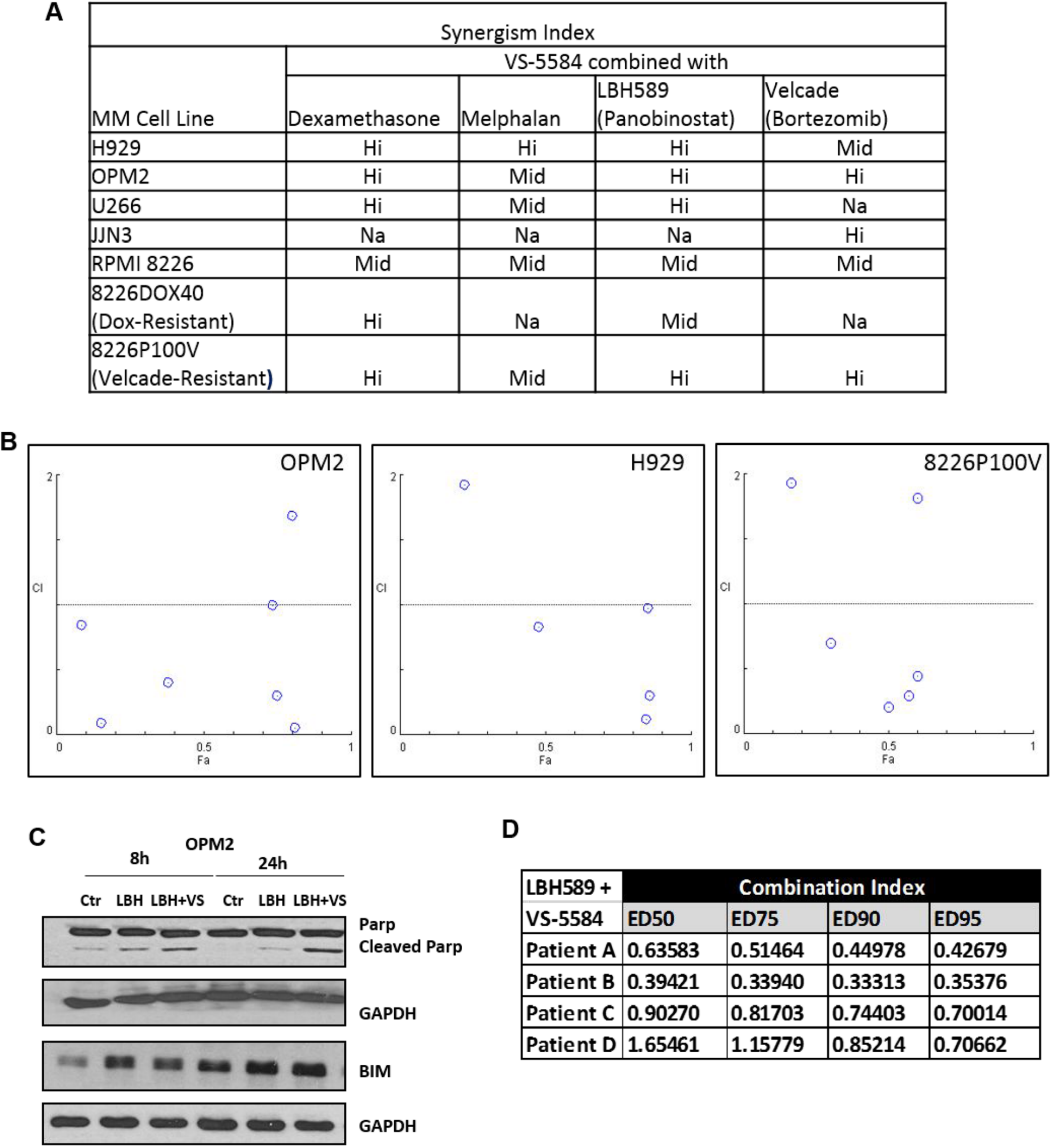
VS-5584 is highly synergistic with Panobinostat in MM cell lines and patient samples (**A**) Multiple Myeloma cell lines were subjected to combination treatment with VS-5584 and Velcade OR Dexamethasone OR Melphalan or Panobinostat for 48 h. Cell viability was then assessed using MTS and the Chou Talalay method utilised to study the synergistic effect of VS-5584 and these drugs in combination. ‘‘Hi’ synergism indicates a synergistic effect at 3 or more concentrations while ‘Mid’ level synergism refers to a synergistic effect at 1–2 concentrations tested. Na indicates the cell line was not tested. (**B**) The Fa-CI plot of 3 selected cell lines H929, OPM2 and Velcade resistant cell line 8226p100V treated with the novel combination of VS-5584 and Panobinostat is shown. (**C**) Apoptosis was analyzed in OPM2 with the combination of (LBH)-Panobinostat (12.3 nM) + (VS) VS-5584 (123 nM) via the assessment of PARP cleavage and Bim levels through immunoblotting. (**D**) CD138 + cells were isolated from bone marrow aspirates from 4 different patients and the combination of Panobinostat and VS-5584 applied over a range of concentrations. Viability was assessed via MTS and combination indices calculated using the Chou-Talalay method. CI < 1 is synergistic CI = 1 is additive and CI > 1 is antagonistic.

A novel drug candidate is more valuable when it is able to synergize with an existing therapeutic making it a likely candidate for clinical trials. We evaluated the efficacy of VS-5584 in combination with Dexamethasone, Melphalan, Velcade and Panobinostat in several MM cell lines. We found that VS-5584 synergized quite broadly across all drug combinations and in different MM subtypes, where the Combination Indices calculated according to the Chou Talalay method yielded values that were less than one (Figure 7A). ‘Hi’ synergism indicates a synergistic effect at 3 or more concentrations while ‘Mid’ level synergism refers to a synergistic effect at 1–2 concentrations tested. The Fa-CI plot of H929, OPM2 and Velcade Resistant MM treated with a combination of VS-5584 and Panobinostat can be studied in Figure 7B. We found this to be a novel and highly synergistic combination not yet described in multiple myeloma. Immunoblotting analysis showed a corresponding increased induction of apoptosis as measured by an elevation in cleaved PARP and pro-apoptotic protein Bim in comparison to single Panobinostat treatment (Figure 7C). In order to elucidate if the efficacy of this novel combination was not limited to cell lines only, we assessed its effects in CD138+ plasma cells freshly isolated from 4 diagnosed MM patients. Interestingly, this drug combination remained highly synergistic in all 4 patients tested over several different doses (Figure 7D). These results propose a novel potent combination of a dual PI3K/mTOR and HDAC inhibitor for the second or third line treatment of relapsed or hard-to-treat myeloma.

## DISCUSSION

A recent study showed that relapsed and refractory multiple myeloma patients had very poor outcomes with an overall survival of 6 months and an event-free survival of 1 month [22]. Thus, it is imperative to develop innovative and novel therapeutic agents so as to improve long-term outcomes especially in patients who derive limited benefit from currently available options.

The PI3K/mTOR/Akt pathway has been implicated to contribute significantly to the pathogenesis of multiple myeloma through the stimulation of proliferation, resistance and cancer stem cell survival [23–26]. Reports show that single agent inhibitors lack significant activity while combinations with lenalidomide, bortezomib and/or dexamethasone do not show meaningful improvement over current clinical combination regimens [27–30]. VS-5584 was designed to increase to overcome PI3K-mediated reactivation of the signaling pathway during mTOR inhibition by inhibiting both targets with equal potency and high selectivity as described earlier (Supplementary Figure 1C). Our previous study has shown that Multiple Myeloma is the most sensitive responder to this dual targeted inhibition which warranted in depth investigation for its novel therapeutic potential.

Indeed VS-5584 is highly efficacious against a large panel of drug-sensitive and drug-resistant MM cell cells as well as in MM patient samples. It seems to trigger anti-myeloma effects by activating a Bim-dependent intrinsic apoptotic pathway. It is noteworthy that VS-5584 was refractory in 2 out of the 4 t(11;14) cell lines tested. This corroborates with a report by Glasford et al, where t(11;14) cells showed less sensitivity to PI-103, as compared to t(4;14) or t(14;16) cell lines. Where cyclin D2 was significantly downregulated by PI-103 in t(4;14) and t(14;16) cells, cyclin D1 in t(4;14) cells were unresponsive. Interestingly, an analysis of 2 different MM patient databases showed that RARRES3 was significantly under-expressed in patients carrying the 11q translocation subtypes. This led us to hypothesize that RARRES3 may play a key role in sensitizing the cells to dual PI3K/mTOR inhibitor induced cell death in MM.

RARRES3 belongs to the HREV107 type II tumour suppressor gene family [31–34]. It maps to the 11q12 chromosomal region and aberrations at 11q region often occur in various types of cancers including lymphoproliferative disorders [16]. The over-expression of RARRES3 has been shown to reverse the tumour phenotype and conversely, RARRES3 expression seems suppressed in several different cancers including B-cell lymphocytic leukemia, breast and colorectal cancer [16, 34–40].

We found a significant dose-dependent increase in RARRES3 mRNA and protein levels in the sensitive H929 and OPM2 but not in the resistant KMS12BM cell line. Furthermore, knocking down RARRES3 significantly reversed cell death triggered by VS-5584 indicating that upregulation of RARRES3 is an important mediator of the anti-myeloma effect of VS-5584.

Since there was a correlation between the expression of levels of RARRES3 and the translocation subtypes t(4;14) and t(11;14) and the fact that these subtypes are defined by overexpression of cyclin D2 and cyclin D1 respectively, we hypothesized that RARRES3 may mediate G1 arrest in MM during VS-5584 treatment by regulating cell cycle proteins. Further support of this hypothesis is provided by a report showing the inhibition of cyclin D protein during RARRES3 overexpression in skin cancer cells [41]. When RARRES3 is knocked down, there is specific upregulation of cyclin D2 but not cyclin D1 or indeed other cyclins. Additionally, we observed an upregulation of CDK4 which is cyclin D target. Taken together, we propose that the increase in RARRES3 mediated by VS-5584 led to the suppression of cyclin D2 and CDK4 and resulted in the induction of G1 arrest followed by apoptosis triggered by VS-5584. Because levels of RARRES3 in KMS12BM or t (11;14) cells were not or could not be regulated by VS-5584, we propose that the regulation of RARRES3 is a critical component of VS-5584 induced cell death in myeloma.

In the list of genes sensitively upregulated by VS-5584 treatment, we were intrigued to find DKK1. Dickkopf-1(DKK1) is a potent inhibitor of the β-catenin pathway. Reports have shown that MM cell lines as well as patient MM samples express high levels of β-catenin not observed in normal plasma cells [19, 20]. This accumulation is attributed likely to posttranslational modifications and evidence so far has not shown any mutation in the signaling partners within the WNT pathway thus indicating that there must be other mechanisms leading to constitutive Wnt activation [19, 20, 42]. β-catenin is involved in the cell cycle regulation, proliferation and invasion of MM cells thus contributing to an oncogenic and metastatic phenotype [19, 21]. We found that VS-5584 resulted in downregulation of active beta-catenin in the sensitive but not resistant cell lines. Recently, a report has shown that VS-5584 can effectively target cancer stem cells as well [43]. The degradation of the β-catenin by VS-5584 may potentially be one of the mechanisms by which cancer stem cells are targeted.

In breast cancer, RARRES3 acts as a tumor suppressor by repressing the Wnt/Beta catenin signaling through the protein deacylation of Wnt/β-catenin signaling molecules [18]. This results in the phosphorylation, ubiquitination and degradation of β-catenin which inhibits cell proliferation, epithelial-mesenchymal transition and stemness (EMT) of breast cancer cells. This also results in the downregulation of β-catenin targets such as cyclin D and survivin. We initially hypothesized that RARRES3 maybe downregulating cyclin D2 through β-catenin. However, we did not observe any significant changes in mRNA or protein expression of β-catenin when RARRES3 was knocked down (data not shown). Nonetheless, when we restored active β-catenin levels with the pre-treatment of LiCl, it significantly prevented cell death triggered by VS-5584. Although LiCl is not a direct activator of β-catenin, the western blot shows that β-catenin levels were indeed restored in tandem with downstream targets of β-catenin and survivin. Ultimately however, a specific β-catenin overexpression may give a more accurate analysis of the role of β-catenin in mediating the mechanism of action of the dual PI3K/mTOR inhibitor. Although RARRES3 may not be downregulating cyclin D through β-catenin, the data shows that the degradation of β-catenin contributes to VS-5584 induced cell inhibition. To our knowledge the involvement of RARRES3 and β-catenin in mediating the effects of dual PI3K/mTOR inhibitors have not been identified before and will contribute further insights to the mechanism of action of these inhibitors in MM as well as other cancers.

Altogether, our data also proposes that RARRES3 may be a useful biomarker for the clinical application of VS-5584 treatment, and analyses of patient databases UAMS and APEX suggest that VS-5584 may be less efficient for MM patients with 11q13 translocations but more successful in patients with maf translocations. In addition, the degradation of β-catenin by VS-5584 in MM, taken together with the report highlighting how VS-5584 preferentially targets cancer stem cells infers that it has the potential to bring about a more durable remission for MM patients especially if used together with a chemotherapeutic drug that diminishes the bulk of the tumor.

VS-5584 is synergistic with clinically therapeutic drugs Dexamethasone and Velcade. The best synergy was evidenced by a novel combination with HDAC inhibitor, Panobinostat. Not only was this novel combination effective in a panel of sensitive and resistant MM cell lines, we also found it efficacious in a series of MM patient samples. Interestingly, synergistic combinations between HDAC inhibitors and inhibitors of the PI3K/mTOR/Akt pathway have recently been observed in pancreatic cancer, B-cell acute lymphoblastic leukemia and Non-Hodgkin's lymphoma [44–46]. This data proposes an exciting potential for the clinical application of the combination VS-5584 and Panobinostat as a novel therapeutic strategy.

Altogether our findings suggest that VS-5584 may provide a more enduring chemotherapeutic treatment response and is potentially a promising clinical candidate for combination treatment in relapsed and refractory patients.

## MATERIALS AND METHODS

### Materials

#### Cell lines and primary patient samples

Human Multiple Myeloma Cell Lines (HMCL) were cultured in RPMI 1640 (Biowest) supplemented with 10% FBS (Biowest) and grown in a humidified atmosphere at 37°C with 5% CO_2._ All HMCL were gifts from Mayo Clinic (Scottsdale, AZ). Mycoplasma testing and cell line authentication via STR profiling were performed in-house. Bone marrow aspirates were collected from MM patients, and CD138^+^ cells purified using CD138 microbeads (Militenyi Biotec) following RBC lysis. Patient samples were cultured in IMDM (GIBCO) and supplemented with 10ng/mL of IL-6 and 100ng/mL of IGF-1 during treatment with VS-5584. Peripheral blood samples were collected from patients and PBMCs isolated using Ficoll centrifugation. Cells were stimulated with 5ng/mL of PHA (Sigma).

#### Chemical compounds

VS-5584 [5-(9-isopropyl-8-methyl-2-morpholin-4-yl-9H-purin-6-yl)-pyrimidin-2-ylamine] was synthesized and developed by S*BIO Ltd and subsequently provided by Verastem Ltd. The other drugs were purchased or donated respectively: Velcade (Millennium Pharmaceuticals), LBH589 [Panobinostat] (Novartis), Melphalan (Sigma), Dexamethasone (kindly provided by Dr Allan Yeoh's lab), Lithium Chloride (Sigma).

### Methods

#### Cell viability

Cell viability was measured by MTS Assay or CTG Assay (Promega Madison, WI). Dose response curves were plotted to determine the IC50 values using the XLFit software (IDBS Ltd). The combination index (CI) is a value derived by the Calcusyn software used to quantitatively describe dose-effect relationships of a combination of 2 different drugs according to the Chou Talaylay method paper [47]. Synergism is defined as a greater than additive effect of the combination of 2 drugs where CI < 1, and additive effect where CI = 1 and an antagonistic effect where CI > 1.

#### Apoptosis and cell cycle

Apoptosis: Cells were processed using an Annexin V/Propidium Iodide (PI) kit (BD Pharmingen). Cell cycle: Cells were stained using a solution of PI and RNase A after fixation with 70% ethanol. Subsequently 10,000 events were collected with the LSRII and analysed with the FlowJo software.

#### Immuno-blotting

Cells were lysed with RIPA, separated on an SDS-PAGE and transferred onto a PVDF membrane. Membrane is then probed with PARP, Bax, Bid, Bim, Bcl-2, Bcl.xL, VDAC, phospho-AKT, phospho-S6 ribosomal protein, phospho 44/42 MAPK, activated β-catenin (Cell Signaling), Beta Catenin, RARRES3, GAPDH (Santa Cruz) antibodies.

#### Isolation of the mitochondrial and cytosolic sample

Samples were fractionated using the Thermo Scientific Mitochondria Isolation Kit for cultured cells via the Dounce homogenization method. Samples were then centrifuged at 20,000g for 15 mins to isolate the mitochondrial pellet. The mitochondrial pellet was then lysed with RIPA lysis buffer.

#### Caspase activity

Caspase activity was measured using the Caspase 3/7-Glo Assay (Promega). 20,000 cells per well were seeded and treated in triplicate with VS-5584. To account for the different number of cells in each well at the end timepoint due to treatment with VS-5584, the protein concentration of each sample was measured and the caspase activity calculated as a function of the relative luminescence measured over protein concentration measured.

#### Microarray

RNA was extracted using the RNeasy Mini Kit according to manufacturer's instructions (Qiagen). RNA quantity and purity were assessed with the use of the NanoDrop (Agilent). Gene Expression profiling (GEP) was performed using the HuGene-1_0-st Affymetrix Human Gene 1.0 ST Array transcript (gene) version (Affymetrix, CA, USA) according to the manufacturer's protocol. This data can be accessed on GEO with accession number GSE84862.

#### Transfection and lentiviral infection

MM cells were transfected each time with siScrambled or siRARRES3 (Dharmacon) with the Neon transfection kit at the settings of 1050mV, 30ms, 1 pulse.

High-titre lentiviruses were generated in HEK293T cells by the transfection of the control (pLKO.puro) or shBIM plasmids together with packaging vectors and pCMV-VSV-G (Sigma) using Xtremegene HP DNA (Roche). 48h after transfection the supernatant containing the viruses were collected and concentrated by centrifugation (Amicon). Infection of MM1.S was performed by spin infection method.

#### Xenograft study

Tumors were induced by the injection of OPM2 cells subcutaneously into the right flank of NOD-SCID female mice. When the tumors were palpable these mice randomly received 11 mg/kg of VS-5584 or its vehicle where each group was *n* = 6. The drug was administered orally on a schedule of 5 days followed by 2 days rest. Mice were monitored regularly for changes in tumor size, weight and signs of infection. Standard protocol was followed in compliance with the guidelines of NIH and NACLAR.

## SUPPLEMENTARY MATERIALS FIGURE


