# Supplementary material for: VS-5584 mediates potent anti-myeloma activity via the upregulation of a class II tumor suppressor gene, RARRES3 and the activation of Bim
Source: Oncotarget. 2017 Oct 20;8(60):101847–64. doi: 10.18632/oncotarget.21988 (PMC5731918; doi:10.18632/oncotarget.21988)
Supplement: Supplementary file 1 [file oncotarget-08-101847-s001.pdf]

# VS-5584 mediates potent anti-myeloma activity via the upregulation of a class II tumor suppressor gene, RARRES3 and the activation of Bim

SUPPLEMENTARY MATERIALS

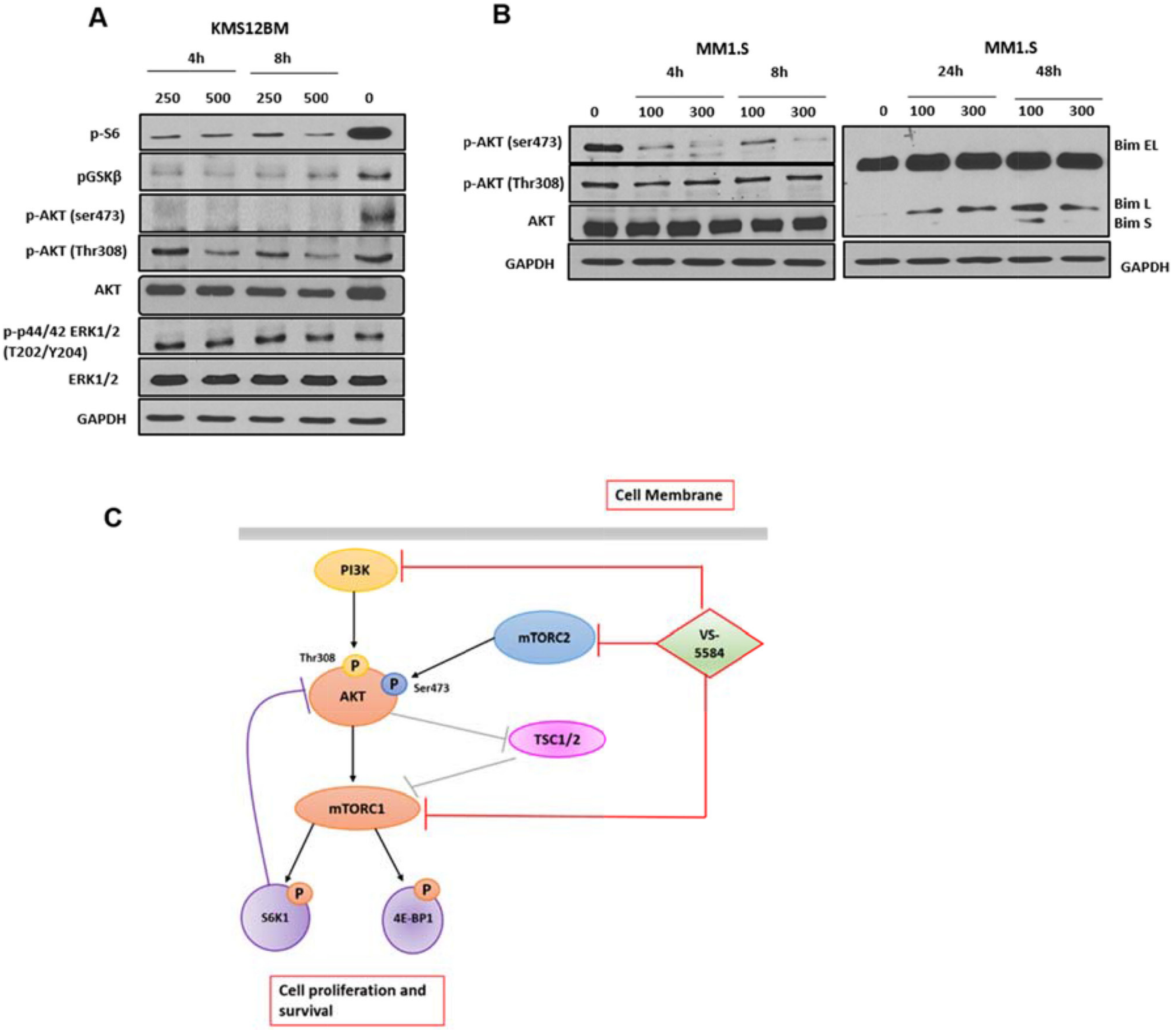

Supplementary Figure 1: (A) VS-5584 can suppress PI3K and mTOR signaling in KMS12BM and MM1.S. KMS12BM was treated VS-5584 for 4 h and 8 h at concentrations indicated. After cell lysis the phosphorylation status of pS6, pAkt, pGSKB and p44/42 MAPK (ERK1/2) were detected by immunoblotting. (B) VS-5584 suppresses phosphorylation of Akt and induces upregulation of Bim in MM1.S. MM1.S cells were treated with VS-5584 at the respective timepoints and concentrations followed by a western blot analyses. (C) Schematic for the dual inhibitory activity of VS-5584 on the PI3K/mTOR/AKT signaling pathway.
